# A Nucleotide Signature for the Identification of American Ginseng and Its Products

**DOI:** 10.3389/fpls.2016.00319

**Published:** 2016-03-18

**Authors:** Yang Liu, Xiaoyue Wang, Lili Wang, Xiaochen Chen, Xiaohui Pang, Jianping Han

**Affiliations:** Institute of Medicinal Plant Development, Chinese Academy of Medicinal Science and Peking Union Medicinal CollegeBeijing, China

**Keywords:** American ginseng, Chinese patent medicine, DNA barcoding, internal transcribed spacer, nucleotide signature

## Abstract

American ginseng (derived from *Panax quinquefolius*) is one of the most widely used medicinal herbs in the world. Because of its high price and increasing demand, there are many adulterants on the market. The proposed internal transcribed spacer 2 (ITS2) has been used to identify raw medicinal materials, but it is not suitable for the identification of Chinese patent medicine ingredients. Therefore, a short barcode for the identification of processed American ginseng and its corresponding Chinese patent medicines would be profitable. In this study, 94 samples of American ginseng and Asian ginseng were collected from all over the world. The ITS2 region was sequenced, and a nucleotide signature was developed based on one single nucleotide polymorphism (SNP) site unique to American ginseng. The nucleotide signature (atcactcctt tgcgggagtc gaggcgg) consists of 27 bases over the length of the ITS2 sequence (420 bp). Furthermore, we also designed primer pairs to amplify the nucleotide signature; the specific primer pair 4F/4R has been found to be unique to the ginseng species and capable of amplifying the nucleotide signatures from Chinese patent medicines and decoctions. We used the nucleotide signature method to inspect ginseng products in Chinese patent medicines; 24 batches of Chinese patent medicine from stores in Beijing were amplified and sequenced successfully. Using the double peaks at the SNP sites of the nucleotide signature, 5 batches were found to be counterfeits, and 2 batches were found to contain adulterants. Thus, this nucleotide signature, with only 27 bp, has broadened the application of DNA barcoding in identification of decoctions, Chinese patent medicines and other ginseng products with degraded DNA. This method can rapidly identify ginseng products and could also be developed as an on-site detection method.

## Introduction

American ginseng (derived from *Panax quinquefolius*) is one of the most widely used medicinal herbs in the U.S. (Ang-Lee et al., 2001) and has been used as a traditional Chinese medicine (TCM) for thousands of years to help attenuate the frequency, severity, and duration of cold and flu symptoms by reinforcing the immune system (Fuzzati, 2004). American ginseng may have potential value for colorectal cancer chemoprevention by the conversion of the parent ginsenosides to bioactive ginseng metabolites (Yu et al., 2015). There are many types of American ginseng products, such as root slices, capsules, powders, and Chinese patent medicines. Over recent years, there has been a growing demand for ginseng products due to the rapid emergence of food therapy in herbal medicine. Asian ginseng and American ginseng are adulterants of each other and share similar morphological and chemical characteristics, but they show different pharmacological properties and medicinal values. It is notoriously difficult to distinguish Asian ginseng and American ginseng using conventional taxonomic methods, especially when they have been processed into Chinese patent medicines or decoctions. One approach is to apply high-performance liquid chromatography-mass spectrometry (HPLC-MS), choosing ginsenosides as markers (Chan et al., 2000). Researchers have extracted at least 30 ginsenosides, including Rb1, Rb2, Rc, Rd, Re, Rg1, and Rg3, from American ginseng (Assinewe et al., 2003), but the profiles of the ginsenosides are similar among the species of this small genus (Chen et al., 2001). Molecular approaches using DNA as markers with phylogenetic analysis have received considerable attention in recent years for the differentiation of closely related species. DNA molecular markers such as RAPD (Shaw and But, 1995; Mihalov et al., 2000; Um et al., 2001; In et al., 2005), ISSR (Bang et al., 2004; In et al., 2005), SSR (Hon et al., 2003; Kim J. et al., 2007), AFLP (Ha et al., 2002), RFLP (Ngan et al., 1999; Kim O. T. et al., 2007), SCAR (Wang et al., 2001; Choi et al., 2008), and ARMS (Park et al., 2006) have been developed for the identification of American ginseng; however, considering the complex ingredients and degraded DNA of Chinese patent medicines, these markers have their own deficiencies. Therefore, a rapid method for precisely analyzing Chinese patent medicine ginseng products is highly desirable.

DNA barcoding, a burgeoning molecular technique utilizing one recognized and relatively short DNA sequence to identify species (Hollingsworth, 2011; Hollingsworth et al., 2011), is a relatively simple and universal tool for non-taxonomist scientists researching plant materials, especially traditional Chinese herbal medicines. DNA barcodes are convenient, highly efficient, and accurate, and thus have a wide range of potential applications in the authentication of medical plants (Hao et al., 2010). As candidate DNA barcodes in the plant kingdom, ITS/ITS2 sequences have been extensively tested and evaluated (Shilin et al., 2010). In our previous study, ITS2 exhibited a 91% PCR recovery efficiency, while ITS only exhibited a 23% recovery efficiency using one primer pair. Based on 12,861 analyzed ITS and ITS2 sequences, we concluded that ITS2 can be used as a mini-barcode to effectively identify species in a wide variety of specimens and medicinal materials (Han et al., 2013). Recently, ITS2 has been widely used for herb identification. Yao et al. (Hui et al., 2010) proposed that ITS2 sequences can be used as universal markers for the identification of plant species and established a website with DNA barcodes for TCMs (http://www.tcmbarcode.cn/) for every researcher's convenience. Zhao et al. used ITS2 to identify Acanthopanacis cortex and its adulterated versions (Zhao et al., 2014). Song et al. used ITS2 to successfully distinguish commercial *Rhodiola* products and found that 60% contained adulterants (Xin et al., 2015). Chen et al. developed two single nucleotide polymorphism (SNP) sites in the ITS2 region that can be used to identify *Panax ginseng* and *Panax quinquefolius* (Xiaochen et al., 2013). This method is suitable for the identification of medicinal materials, slices, seeds and powders, but it is unsuitable for the identification of heavily processed materials such as decoctions and Chinese patent medicines because of the DNA degradation occurred in the manufacturing processes. The DNA degradation that occurs in processed biological material often prevents the recovery of PCR fragments longer than 200 bp (Goldstein and Rob, 2003; Hajibabaei et al., 2006; Wandeler et al., 2007). Meusnier et al. proposed the use of a “mini-barcode” sequence to overcome this obstacle. They cut the full length *CO1* into different lengths; while the 650-bp length could distinguish 97% of the species tested, the short lengths of 150 bp and 100 bp achieved 95% and 90% identification success, respectively (Meusnier et al., 2008). Shaw et al. (Lo et al., 2015) successfully obtained an 88-bp fragment from TCM material after it had been boiled for 120 min, but the amplification of a 121-bp fragment was unsuccessful, indicating that shorter degraded DNA fragment lengths are more likely to be amplified. The phrase “nucleotide signature” refers to one or more nucleotides unique to one taxon, and a nucleotide signature has been used in the identification of *Aglaia stellatopilosa* (Ng et al., 2014). These considerations motivated our group to develop a short nucleotide signature within the ITS2 region.

This study aimed to develop a short gene identifier for the differentiation of American ginseng and its Chinese patent medicines. A 27-bp (atcactcctt tgcgggagtc gaggcgg) gene identifier was found. This exclusive nucleotide signature of American ginseng is a section of short motifs that is well conserved within the species. The developed method was later used for the analysis of ginseng products, including 24 batches of Chinese patent medicine. Detailed information is below.

## Materials and methods

### Collection of materials

A total of 94 samples of American ginseng and Asian ginseng were collected by our group from the following locations: Muling City; Ning'an City, Heilongjiang Province; Ji'an City; Tonghua City; Jingyu County; Fusong County, Jilin Province; Xinbin county; Qingyuan county, Liaoning Province; the Beijing Huairou area; Wendeng City, Shandong Province; the Anguo herb market in Hebei Province; the Bozhou herb market in Anhui Province; Canada; and Wisconsin, United States of America. All the samples were authenticated by Prof. Lin Yulin at the Institute of Medicinal Plant Development (IMPLAD). The details of these samples are listed in Table 1S. We also downloaded from GenBank all 283 complete ITS2 sequences of the *Panax* species that can be confused with *P. quinquefolius* according to their morphological characteristics. These sequences were used as the reference for development of the American ginseng nucleotide signature. For further study, 24 batches of Chinese patent medicines containing ginseng products were purchased from stores in Beijing.

### DNA extraction, amplification, and sequencing

#### Root or powder

Each root sample was sterilized by being scrubbed with 75% ethanol and was then ground by using a ball-milling machine (Retsch, Germany); 20 mg of each ground sample was subsequently used for the extraction of genomic DNA. The isolation of DNA was performed with the Plant Universal Genomic DNA Kit (Tiangen Biotech Beijing Co., China) according to the manufacturer's instructions. The primer pair 2F (ATGCGATACTTGGTGTGAAT)/3R (GACGCTTCTCCAGACTACAAT) diluted to 2 μmol/μL was used for the amplification of ITS2 (Shu-Jiau et al., 2007). PCR was performed in a 25-μL reaction system containing 2 μL template DNA, 2.5 μL PCR Buffer (10 ×), 2 μL Mg^2+^ (25 mmol/L), 2 μL dNTP mixture (2.5 mmol/L, 1.0 μL primers 2F/3R (2.5 μmol/L), and 1.0 U Taq DNA polymerase. The conditions for PCR were 40 cycles at 94°C for 30 s, 56°C for 30 s, and 72°C for 45 s. The PCR process was ended by 10 min of incubation at 72°C. The purified PCR products were sequenced by the Major Engineering laboratory of the Chinese Academy of Agricultural Sciences University with the primer pair 2F/3R. All the sequences underwent forward and backward sequencing.

#### Decoction and chinese patent medicine

American ginseng powder (20 g) was boiled in 500 mL double-distilled water for 90 min. The volume of the decoction system was maintained at 500 mL throughout the boiling process by the addition of double-distilled water, and the supernatant of the resulting decoction was used for DNA extraction. The DNA from the decoction supernatant was extracted with the Plant Universal Genomic DNA Kit (Tiangen Biotech Beijing Co., China) according to the manufacturer's instructions. DNA from the Chinese patent medicine was extracted by cryogenic grinding and use of the Plant Universal Genomic DNA Kit (Tiangen Biotech Beijing Co., China). The degraded DNA was amplified using five primer pairs newly designed using Primer 6.0 software (Glantz, 2005). Details of these primers are shown in Table 1. The conditions for PCR were 40 cycles of 94°C for 30 s, 55°C for 30 s, and 72°C for 45 s. PCR was performed in the same 25-μL reaction system that was used for the other samples. The purified PCR products were sequenced by the Major Engineering laboratory of the Chinese Academy of Agricultural Sciences University with the newly designed primer pairs.

**Table 1:** **Primers used for PCR amplification and sequencing**.

**Primer pair**	**Primer name**	**Direction**	**Primer sequences (5′−3′)**	**Amplicon size**	**Annealing temperature**
1	2F	Forward	ATGCGATACTTGGTGTGAAT	420	56
	3R	Reverse	GACGCTTCTCCAGACTACAAT		
2	11F	Forward	CCCAAATGCGAGTCCTTG	49	55
	12R	Reverse	TTACAACCACCACTTGTCG		
3	11F	Forward	CCCAAATGCGAGTCCTTG	72	55
	11R	Reverse	GCACGACATGAGAAGAGG		
4	6F	Forward	CCCAACCCATCACTCCTT	95	55
	4R	Reverse	GCCAAGGACTCGCATTTG		
5	7F	Forward	CCCAACCCATCACTCCTT	149	55
	7R	Reverse	CGCACGACATGAGAAGAG		
6	4F	Forward	TGCAGAATCCCGTGAACC	168	55
	4R	Reverse	GCCAAGGACTCGCATTTG		

### Sequence assembly and alignment

The sequences were assembled using CodonCode Aligner 3.7.1 (CodonCode Co., Germany). We first removed low-quality motifs at both ends of the sequencing results. Then, all the ITS2 sequences of the *Panax* genus, including those downloaded from GenBank, were annotated and delimited using a hidden Markov Model (HMM)-based method (Keller et al., 2009) to eliminate the 5.8S and 28S rDNA regions. The haplotypes of every *Panax* species were selected by CodonCode Aligner. All the haplotypes were aligned using MEGA 5 software (Tamura, 2011).

## Results

### Development of a nucleotide signature for *P. quinquefolius*

The PCR amplification and sequencing success rates of the ITS2 region were 100% using the primer pair 2F/3R. The PCR amplification of all 94 samples produced sequences 420 bp long. A total of 54 ITS2 sequences of American ginseng generated from experimental materials and retrieved from GenBank were aligned. The results showed that the ITS2 regions of all the individuals within the *P. quinquefolius* species were completely conserved. No variable sites were found among them. In accordance with previous studies (Xiaochen et al., 2013), two SNPs in ITS2 were found to exist stably between American ginseng and Asian ginseng. In the two SNPs, the nucleotide (T) at position 32 was found to be unique to *P. quinquefolius*. Based on the SNP site, we developed a 27-bp nucleotide signature (atcactcctt tgcgggagtc gaggcgg) for American ginseng.

### Validate the nucleotide signature for *P. quinquefolius*

**Figure 1:**
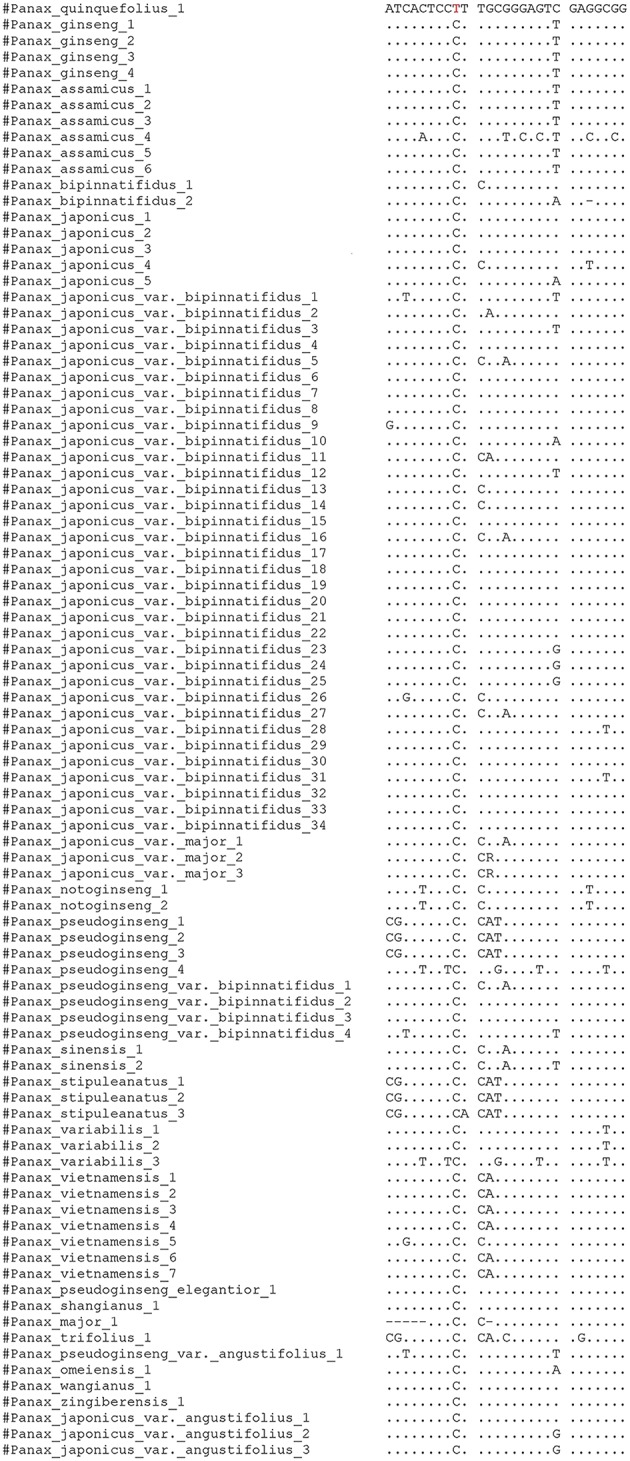
**Result of a nucleotide blast of the 27-nucleotide conserved region**.

**Figure 2:**
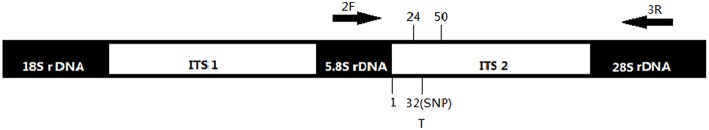
**Schematic diagram showing the nucleotide signature region of *P. quinquefolius***.

All the haplotypes representing the 283 ITS2 sequences of the closely related *Panax* species (downloaded from NCBI) were aligned using MEGA 5, and a summary is shown in Figure 1. In this conserved region of American ginseng, other species have 1–7 divergent nucleotides. Therefore, this region is defined as the nucleotide signature of *P. quinquefolius*. The BLAST results showed that the conserved 27-bp nucleotide region can be used to differentiate *P. quinquefolius* from all the other *Panax* species retrieved from the NCBI database. The details of this nucleotide signature are shown in Figure 2.

### Selection of a species-specific primer pair for the amplification the nucleotide signature

**Figure 3:**
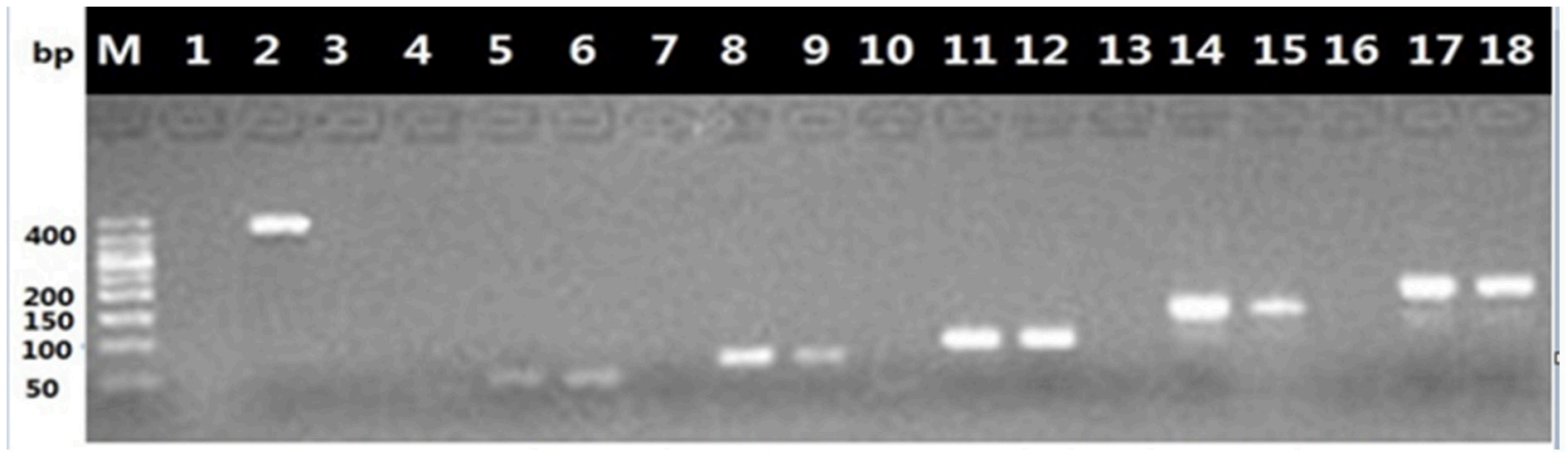
**Six primer pairs amplify the different sizes of sequences from decoction**. Except for the first lane (Marker), each primers pairs takes up three lanes. Lanes from left to right is 2F/3R, 11F/12R, 11F/11R, 6F/4R, 7F/7R, and 4F/4R. The first lane in each primers pair group (lane 1, 4, 7, 10, 13, and 16) is negative control. The second lane (lane 2, 5, 8, 11, 14, and 17) is positive control. The third lane (lane 3, 6, 9, 12, 15, and 18) is PCR products from decoction.

We used the American ginseng decoction to verify whether the nucleotide signature method functions with processed materials. The complete ITS2 region (420 bp) could not be amplified from the decoction using universal primer pair 2F/3R. Thus, we designed five primers for nucleotide signature amplification by aligning ITS2 sequences and identifying conserved regions ranging from 50 to 200 bp in size. Visible PCR products were obtained (Figure 3). The five PCR products were 49 bp, 72 bp, 95 bp, 149 bp, and 168 bp long. The results showed that although the entire 420-bp ITS2 region could not be amplified, the shorter barcode can still be amplified from the degraded DNA. Two of the primer pairs (6F/4R and 7F/7R) were designed at the SNP sites, thus the nucleotide signature could not be obtained after sequencing and truncated the primer region. Two other designed primer pairs (11F/11R and 11F/12R) successfully amplified the shorter length amplicon, but the PCR product was too short to be sequenced. The primer 4F/4R successfully amplified the nucleotide signature, and the sequencing results demonstrated that the short nucleotide signature was successfully obtained from the American ginseng decoction.

### Validation of the nucleotide signature for the identification of Chinese patent medicines

**Figure 4:**
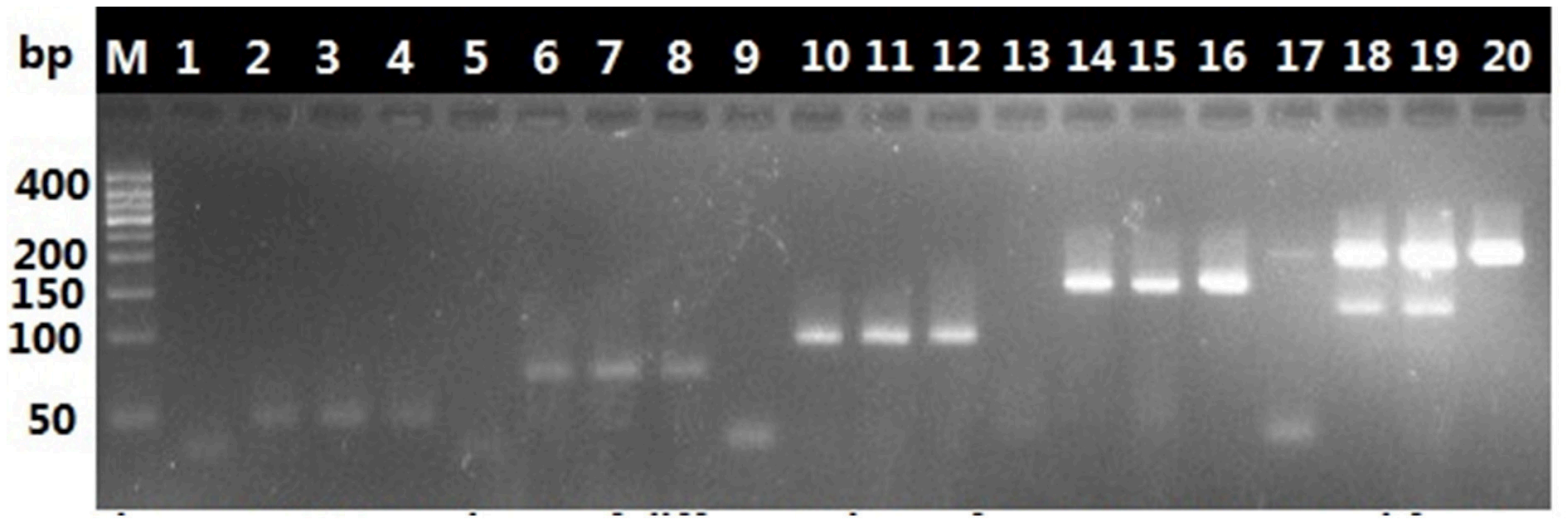
**Six primer pairs amplify the different sizes of sequences from Yangshen Baofei Pills**. Except for the first lane (Marker), each primers pairs takes up four lanes. Lanes from left to right is 11F/12R, 11F/11R, 6F/4R, 7F/7R, and 4F/4R. The first lane in each primers pair group (lane 1, 5, 9, 13, and 17) is negative control. The fourth lane (lane 4, 8, 12, 16, and 20) is positive control. The other two lanes are PCR products from Yanshen Baofei Pills.

The American ginseng in Chinese patent medicines goes through various processes that make the authentication of commercial products difficult. Yangshen Baofei Wan is a representative Chinese patent medicine that contains American ginseng in addition to 11 other ingredients. The complete ITS2 region (420 bp) could not be amplified from this Chinese patent medicine using the universal primer pair 2F/3R. We tested the ability of the 4F/4R primer pair to amplify the nucleotide signature region from this Chinese patent medicine. The results showed that the targeted nucleotide signature of American ginseng was successfully amplified from the 2 batches of Yangshen Baofei Wan that were tested (shown in Figure 4). This positive result led to the following examination of other Chinese patent medicines containing ginseng products.

To validate this method, 22 batches of other Chinese patent medicines containing American ginseng, Asia ginseng and notoginseng were tested. The results showed that the primer pair 4F/4R was very specific to *Panax* species. The PCR and sequencing success rate was 100%. No Asian ginseng adulterates were found in 1 batch of Xiyangsheng pills, 2 batches of Xiyangsheng tablets and 2 batches of Xiyangsheng capsules from different manufacturers. They were all found be made with genuine American ginseng. Most Chinese patent medicines contain different types of species. For example, Dieda pills contain 24 ingredients, including Notoginseng (Sanqi), *Angelicae Sinensis Radix* (Danggui), *Paeoniae Radix Alba* (Baishao), *Paeoniae Radix Rubra* (Chishao), *Persicae Semen* (Taoren), *Carthami Flos* (Honghua), *Draconis Sanguis* (Xuejie), *Siphonostegiae Herba* (Beiliujinu), *Drynariae Rhizoma* (Gusuibu), *Dipsaci Radix* (Xuduan), *Sappan Lignum* (Sumu), *Moutan Cortex* (Mudanpi), *Olibanum* (Ruxiang), *Myrrha* (Moyao), *Curcumae Longae Rhizoma* (Jianghuang), *Sparganii Rhizoma* (Sanleng), *Saposhnikoviae Radix* (Fangfeng), *Melo Semen* (Tianguazi), *Aurantii Fructus Immaturus* (Zhishi), *Platycodonis Radix* (Jiegeng), *Glycyrrhizae Radix et Rhizoma* (Gancao), *Akebiae Caulis* (Mutong), *Pyritum* (Zirantong), and *Eupolyphaga Steleophaga* (Tubiechong). The primer pair 4F/4R could specifically amplify the sequence of notoginseng from this Chinese patent medicine. Direct sequencing of the PCR products showed very clean traces. Another example is the Qipi pill. Except for Asian ginseng, there are 10 ingredients in this Chinese patent medicine, and the 4F/4R primer pair also specifically amplified the sequence of this Asian ginseng. Thus, the 4F/4R primer pair exhibited specificity for ginseng products.

**Figure 5:**
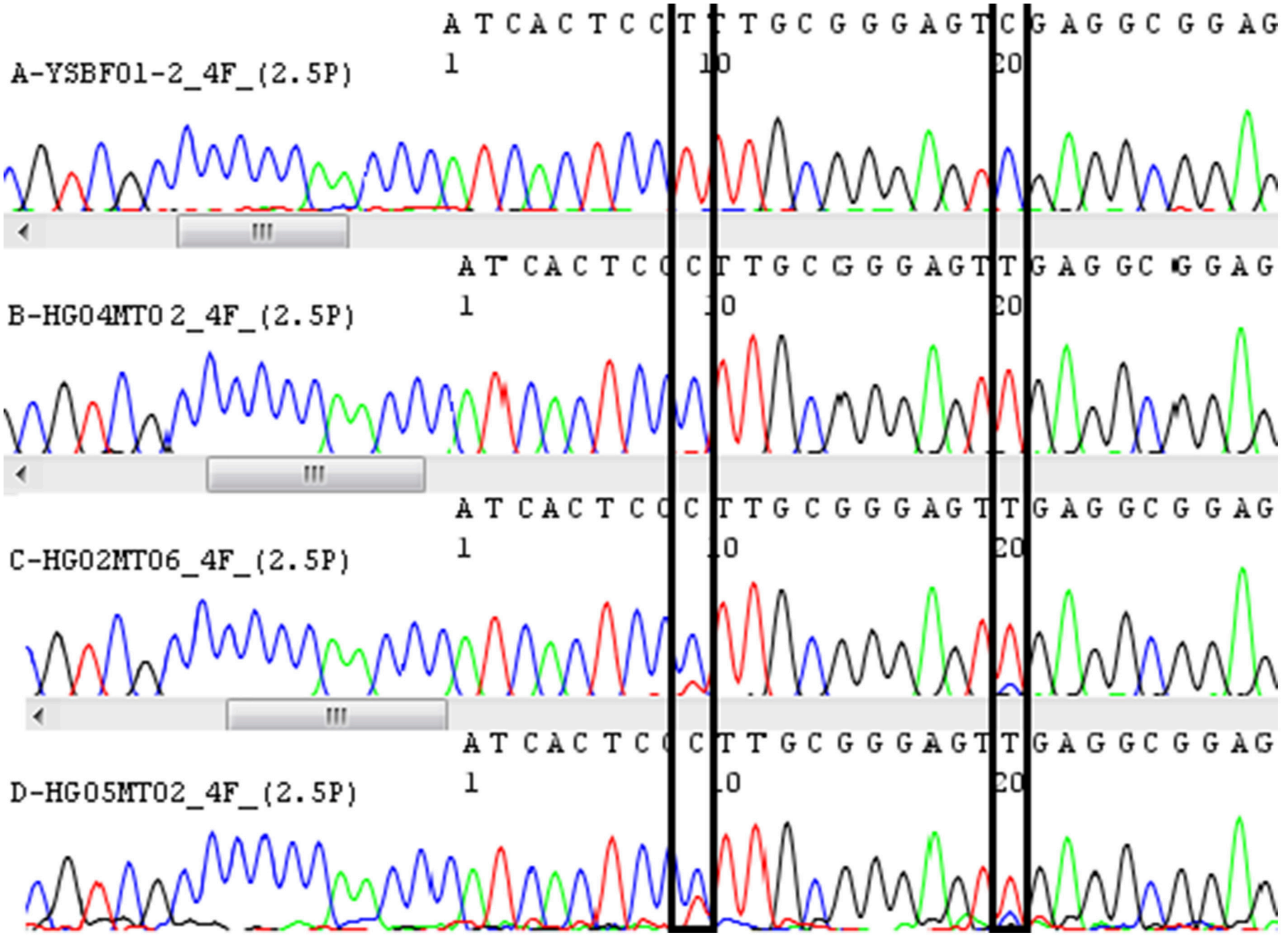
**Peak profile of four batches of Chinese patent medicine labeled containing *Panax* species**. A-YSBF01 represents Yangsheng baofei Pills. B-HG04MT02 represents Renshen Guipi Pills. C-HG02MT06 represents Qipi Pills. D-HG05MT02 represents Shenlin Baizhu Capsules. Two SNP sites were framed respectively in this peak profile. Different bases were represented by peaks of four different colors. Double peaks are clearly examined at SNP sites in Qipi Pills (C-HG02MT06) and Shenlin Baizhu Capsules (D-HG05MT02).

By seeking the nucleotide signature developed in this study, 5 of 21 Chinese patent medicines labeled as containing Asian ginseng were found to contain American ginseng instead. These were 2 batches of Shenwu Jiannan capsules (HG08MT01, HG08MT02), 1 batch of Renshen Guipi pills (HG04MT01) and 2 batches of Qipi pills (HG02MT03, HG02MT05) from different manufacturers. The clean traces showed that there was no Asian ginseng detected in these five counterfeits. By analyzing the double peak at the SNP positions (Figure 5), 2 batches were found to be adulterated with American ginseng (HG02MT06, HG05MT02). The other 17 Chinese patent medicines that listed ginseng products as ingredients were found to match their labels.

## Discussion

### Why develop a nucleotide signature to identify Chinese patent medicine ingredients

Chinese patent medicines have become commonly prescribed and can be easily purchased worldwide. However, the standardization of Chinese patent medicines has still not been established, and the composition and quality of those medicines and healthcare products are not guaranteed (Wang et al., 2011), which can cause disorder in the medicine market. The supervision of the Chinese patent medicine market is made even more difficult by the lack of effective standardization methods.

As a precious herb, American ginseng has often been processed into different types of products, and heat-processed American ginseng roots have been reported be more bioactive than unprocessed roots (Kim K. et al., 2007). Air-dried ginseng produces weak anti-proliferative effects, whereas steamed ginseng exhibits a significant increase in anti-proliferative and pro-apoptotic effects (Sun et al., 2011). However, it is very difficult to identify these processed materials due to ingredient DNA degradation. In this case, traditional barcodes such as ITS and ITS2 are ineffective, and a short reliable marker is required. It has been reported that the smaller the size of the degraded DNA, the higher the amplification success rate will be (Särkinen et al., 2012). This has been demonstrated by a recent study, where a TCM material was boiled for 120 min, and DNA fragments of 88 bp were amplified, but a 121-bp fragment was not (Lo et al., 2015). Therefore, it is recommended to develop conditions for producing very short amplicons for the heavily damaged DNA present in Chinese patent medicines. Therefore, in this study, we developed a 27-bp nucleotide signature for American ginseng.

A nucleotide signature should be identical in all the individuals within a given species; i.e., no mutations are allowed in a nucleotide signature. In this study, a total of 54 ITS2 sequences were used to develop the nucleotide signature of American ginseng, including 40 samples derived from main producing areas and 14 samples generated from different institutes around the world. Furthermore, the results show that different endemic American ginseng samples share the same characteristic signature. All the 283 ITS2 sequences of the *Panax* genus from GenBank were utilized to verify the nucleotide signature. Thus, the data was sufficiently broad to be considered representative. By BLAST analysis, we found that the nucleotide signature was not present in other *Panax* species and non-*Panax* genera. Thus, the nucleotide signature developed in this study can be used as a “gene identifier” for American ginseng. Even the closest related ginseng species has two loci that differ from the *P. quinquefolius* signature. Any sample from which the 27-bp signature sequence (atcactcctt tgcgggagtc gaggcgg) is amplified can be identified as American ginseng.

### The nucleotide signature effectively identifies American ginseng and ginseng products

American ginseng is mainly used to tonify “qi” and nourish “yin,” and Asian ginseng is known to reinforce “qi” and benefit the spleen and the lungs. Because American and Asian ginseng demonstrate different pharmacological properties and medicinal values, they should be clearly distinguished prior to use in Chinese patent medicines. As shown in Table 2, out of the 24 batches of Chinese patent medicines tested, 5 that were supposed to contain Asian ginseng were found to contain American ginseng. In China, all Chinese patent medicines of the same name have the same proportions of ingredients and are manufactured in accordance with the PRC Pharmacopeia's monograph for that particular formula. If the detected ingredients are not consistent with the label, that particular Chinese patent medicine is considered to be a counterfeit. In this study, the percentage of counterfeit samples identified out of the 24 commercial Chinese patent medicine samples was 21%, including 2/2 of the Shenwu Jiannan capsules, 1/3 of the Renshen Guipi pills, and 2/7 of the Qipi pills. In a previous study, a mixed powder of *P. ginseng* and *P. quinquefolius* could be clearly identified by analysis of the double peaks at the SNP positions, and American ginseng contamination of Asian ginseng as low as 5% could be detected (Xiaochen et al., 2013). In this study, by seeking the double peaks at the SNP sites, we detected 2 batches of Chinese patent medicine that were adulterated with American ginseng. The specific primer pair 4F/4R developed in this study could be used to amplify the nucleotide signature region and to identify the ginseng product used in Chinese patent medicines.

**Table 2:** **List of Chinese patent medicine used in this study**.

**Voucher number**	**Name of Chinese patent medicine**	**Stores**	**Listed *Panax* ingredients in label**	**Blast results**
YSBF1	Yangsheng baofei pills	Stores in Beijing	*P. quinquefolius*	*P. quinquefolius*
YSBF2	Yangsheng baofei pills	Stores in Beijing	*P. quinquefolius*	*P. quinquefolius*
HG01MT01	Xiyangsheng pills	Stores in Beijing	*P. quinquefolius*	*P. quinquefolius*
HG01MT02	Xiyangsheng tablet	Stores in Beijing	*P. quinquefolius*	*P. quinquefolius*
HG01MT09	Xiyangsheng capsules	Stores in Beijing	*P. quinquefolius*	*P. quinquefolius*
HG01MT13	Xiyangsheng tablet	Stores in Beijing	*P. quinquefolius*	*P. quinquefolius*
HG01MT16	Xiyangsheng capsules	Stores in Beijing	*P. quinquefolius*	*P. quinquefolius*
HG02MT01	Qipi pills	Stores in Beijing	*P. ginseng*	*P. ginseng*
HG02MT02	Qipi pills	Stores in Beijing	*P. ginseng*	*P. ginseng*
HG02MT03	Qipi pills	Stores in Beijing	*P. ginseng*	*P. quinquefolius*
HG02MT04	Qipi pills	Stores in Beijing	*P. ginseng*	*P. ginseng*
HG02MT05	Qipi pills	Stores in Beijing	*P. ginseng*	*P. quinquefolius*
HG02MT06	Qipi pills	Stores in Beijing	*P. ginseng*	*P. ginseng*
HG02MT07	Qipi pills	Stores in Beijing	*P. ginseng*	*P. ginseng*
HG03MT02	Renshen guipi pills	Stores in Beijing	*P. ginseng*	*P. ginseng*
HG04MT01	Renshen guipi pills	Stores in Beijing	*P. ginseng*	*P. quinquefolius*
HG04MT02	Renshen guipi pills	Stores in Beijing	*P. ginseng*	*P. ginseng*
HG05MT01	Shenlin baizhu capsules	Stores in Beijing	*P. ginseng*	*P. ginseng*
HG05MT02	Shenlin baizhu capsules	Stores in Beijing	*P. ginseng*	*P. ginseng*
HG07MT01	Shenlin baizhu pills	Stores in Beijing	*P. ginseng*	*P. ginseng*
HG07MT04	Shenlin baizhu pills	Stores in Beijing	*P. ginseng*	*P. ginseng*
HG08MT01	Shenwu jiannan capsules	Stores in Beijing	*P. ginseng*	*P. quinquefolius*
HG08MT02	Shenwu jiannan capsules	Stores in Beijing	*P. ginseng*	*P. quinquefolius*
HG09MT02	Dieda pills	Stores in Beijing	*P. notoginseng*	*P. notoginseng*

American ginseng is known to be more expensive than Asian ginseng. By investigating the medicinal market, we found that the explanation for why manufacturers adulterate or substitute Asian ginseng with American ginseng lies in the sales patterns of the different ginseng products. Normally, Asian ginseng is sold in the form of a root with its fibrils, while the roots and fibrils of American ginseng are sold separately, with the fibrils set at a lower price. The counterfeiters purchase American ginseng fibrils to substitute for Asian ginseng to produce Chinese patent medicines. For example, the Qipi pill, a representative Chinese patent medicine, is mainly examined for Asian ginseng, according to the PRC Pharmacopeia, by using a microscope to check whether it contains calcium oxalate cluster crystals. However, American ginseng and Asian ginseng share similar microscopic characteristics, therefore consumers are unable to judge whether the Chinese patent medicine is a counterfeit or adulterated with American ginseng. Due to the lack of an identification method, counterfeit drug incidents have been dramatically increasing over recent years. Every week, new cases of counterfeit medicine are reported around the world (Dégardin et al., 2014). In this study, we collected 7 batches of Qipi pills from different manufacturers. 2 of them were identified as counterfeitsand and 1 of them was discovered to be adulterated with American ginseng using this nucleotide signature method. This method has been demonstrated to be effective for the detection of ginseng products in Chinese patent medicines.

### The 27-bp nucleotide signature dramatically broadens the applications of DNA barcoding for market supervision

One important application of the nucleotide signature lies in obtaining sequence information from Chinese patent medicines and degraded biological materials. Due to the degree of processing of these materials, it is very difficult to amplify relatively long sequences. Indeed, many ginseng products appearing in the market have been boiled, which undoubtedly leads to the degradation of DNA. In the manufacturing of Chinese patent medicines, there are many types of TCM materials that undergo different processing techniques. Thus, the method developed in this study had widely application in authenticate ginseng products contained in these medicines.

For the first time, the nucleotide signature (atcactcctt tgcgggagtc gaggcgg) is proposed for the identification of American ginseng. The nucleotide signature, a type of molecular marker that is more specific than a DNA barcode, is a very efficient tool for the identification of American ginseng and ginseng products. The nucleotide signature can be detected without regard to the sample type or cultivation site. Our group has developed several nucleotide signatures for different types of TCMs, such as Notoginseng (Sanqi) and *Angelicae Sinensis Radix* (Danggui). The nucleotide signature can be successfully amplified from natural plants, root slices, powder products, decoctions and Chinese patent medicines.

Because the price of American ginseng is very high, there are also other adulterants, including *Angelicae Dahuricae Radix* (Baizhi), *Adenophorae Radix* (Nanshashen), and *Platycodonis Radix* (Jiegeng), which exist in the domestic and international markets. These adulterants belong to different families, and the sequence divergences for this nucleotide signature region are very large, which further confirms the specificity of this signature for American ginseng. The nucleotide signature is sufficient and necessary for the identification of American ginseng in Chinese patent medicines. The developed method is rapid, accurate, reliable and highly sensitive for the identification of ginseng products. Therefore, the nucleotide signature, with only 27 bp, has demonstrated promise for the application of the detection of ginseng products in Chinese patent medicines.

However, to distinguish all the ingredients in complex Chinese patent medicines, we recommend the high-throughput sequencing method, which uses an emulsion PCR approach to simultaneously amplify several thousand 100–200-bp DNA molecules in one reaction and yields a large number of short sequences at a lower cost than standard approaches. For the further study of Chinese patent medicine ingredients, a promising direction would be to combine the short nucleotide signature method with the high-throughput sequencing method.

## Conclusion

In this study, we found a 27-bp nucleotide signature within the ITS2 region that can be uniquely detected in American ginseng and its products. Except for American ginseng, none of the other species displayed this nucleotide signature. The nucleotide signature method dramatically broadens the applications of DNA barcoding in Chinese patent medicine. The 4F/4R primer developed in this study could specifically amplify the nucleotide signature region from Chinese patent medicines and decoctions. Further studies on the application of nucleotide signatures to other valuable and precious species would undoubtedly be promising and beneficial.

## Author contributions

JH conceived the study and participated in its design. JH, XC, FS, contributed samples and carried out the experiments. YL analyzed the data and drafted the manuscript. All authors have read and approved the final manuscript.

### Conflict of interest statement

The authors declare that the research was conducted in the absence of any commercial or financial relationships that could be construed as a potential conflict of interest.
